# Acute Myelogenous Leukemia with the t(7;7)(p15;p22) Translocation, a Novel Simple Variant of t(7;11)(p15;p15) Translocation: First Description

**DOI:** 10.1155/2021/5529669

**Published:** 2021-04-19

**Authors:** Motoharu Shibusawa

**Affiliations:** IMS Group, Shinmatsudo Central General Hospital, Department of Hematology, Chiba, Japan

## Abstract

The t(7;11)(p15;p15) translocation is a recurrent genetic abnormality associated with acute myelogenous leukemia (AML). The translocation results in a fusion between the nucleoporin 98 and homeobox genes. We describe a case of AML with t(7; 7)(p15;p22) translocation, which is a novel simple variant of the t(7;11)(p15;p15) translocation. A 66-year-old woman presented with subcutaneous hemorrhage in both forearms. Laboratory test revealed hyperleukocytosis (white blood cell count: 97,800 cells/*µ*L (blasts, 51.0%)), anemia (hemoglobin level: 7.6 g/dL), thrombocytopenia (platelet count: 6.5 × 10^4^/*μ*L), and hyperfibrinolysis (elevated d-dimer level: 12.4 *µ*g/mL; fibrin/fibrinogen degradation products: 26.9 *µ*g/mL). The patient was diagnosed with AML; the blast morphology was unclassifiable according to the French-American-British classification. Flow cytometry CD45 gating revealed that the blasts expressed CD34, CD13, CD33, and CD117. G-banding of tumor cells revealed the t(7;7)(p15;p22) translocation [20/20]. The patient underwent chemotherapy. At 48 days of admission, the patient died of multiple organ failure. The t(7;7)(p15;p22) translocation involved chromosome 7p15, indicating its association with the homeobox genes. To the best of our knowledge, this is the first report of a patient with AML with the t(7;7)(p15;p22) translocation, which is a simple novel variant of the t(7;11)(p15;p15) translocation.

## 1. Introduction

The recurrent genetic abnormalities in acute myeloid leukemia (AML) are associated with distinct clinicopathological characteristics and have prognostic significance. Most of these structural chromosomal rearrangements lead to the creation of a fusion gene encoding a chimeric protein. Many diseases that result from such alterations have characteristic morphological and immunophenotypic characteristics [1]. The t(7;11)(p15;p15) translocation is a recurrent genetic abnormality associated with AML. The translocation results in a fusion between the nucleoporin 98 (NUP98) and homeobox (HOX) genes [2]. The frequency of AML with this translocation is only 2.2% [3], with most cases often reported in Asian countries [3–5]. Compared with patients with AML without the t(7;11)(p15;p15) translocation, the clinicopathological characteristics of those with the translocation are as follows: younger age, predominance in women, and M2-subtype according to the French-American-British (FAB) classification. Moreover, the overall survival (OS) and disease-free survival (DFS) of the latter patient group are poorer than those of the former patient group [3]. A simple variant of the t(7;11)(p15;p15) translocation has been reported [6, 7]. This article describes a novel case of AML with the t(7; 7)(p15;p22) translocation and a literature review of the t(7;11)(p15;p15) translocation.

## 2. Case Presentation

**Figure 1:**
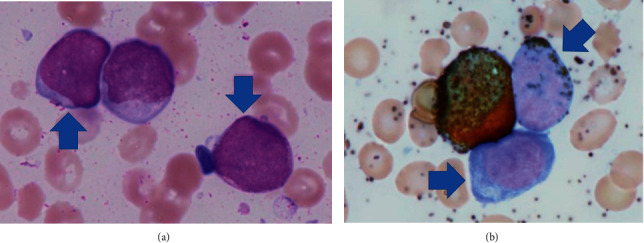
Bone marrow aspiration. (a) Wright–Giemsa staining (×1,000) and (b) myeloperoxidase staining (×1,000). Arrows indicate blasts. Blasts presented with loose reticular chromatin and a high nucleus-to-cytoplasm ratio. Their size ranged from medium to large, and some showed cytoplasmic nucleoli and granules. Most blasts were myeloperoxidase negative.

**Figure 2:**
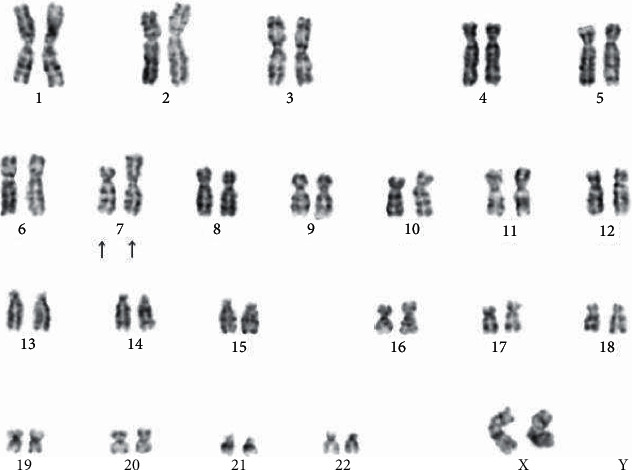
G-banding: translocation t(7;7)(p15;p22).

A 66-year-old Japanese woman visited a local hospital with the chief complaint of malaise for two days. She presented with subcutaneous hemorrhage in both forearms; therefore, she was referred to the hematology department of our hospital. She had no remarkable medical history except pneumonia. Physical examination revealed a body temperature of 37.1°C, blood pressure of 136/86 mmHg, pulse rate of 85 beats per minute, and oxygen saturation of 99% in ambient air. She had no palpable lymphadenopathy, and chest auscultation revealed no rales or murmur. Her abdomen was soft, without palpable hepatosplenomegaly. Laboratory test results were as follows: hyperleukocytosis (white blood cell count: 97,800 cells/*µ*L), differential count (blasts, 51.0%; neutrophils, 31.5%; monocytes, 2.5%; metamyelocytes, 5.5%; lymphocytes, 5.0%; basophils, 4.5%), anemia (hemoglobin level: 7.6 g/dL), thrombocytopenia (platelet count: 6.5 × 10^4^/*μ*L), and hyperfibrinolysis (elevated d-dimer level: 12.4 *µ*g/mL; fibrin/fibrinogen degradation products: 26.9 *µ*g/mL; elevated lactate dehydrogenase level, 1,398 IU/L). According to the bone marrow examination, blasts accounted for 30.4% of cells and presented with delicate nuclear folds and a high nucleus-to-cytoplasm ratio. The size of the blasts ranged from medium to large, and some presented with cytoplasmic nucleoli and granules. Most blasts were myeloperoxidase negative, although some were positive (Figure 1). We were unable to morphologically classify blasts using the FAB classification. Flow cytometry CD45 gating revealed that the blasts expressed CD34, CD13, CD33, and CD117. G-banding of tumor cells and T cells in peripheral blood samples revealed the t(7;7)(p15;p22) translocation [20/20] (Figure 2) and 46, XX [20/20], respectively; thus, the novel translocation was observed. She was diagnosed with AML and the t(7;7)(p15;p22) translocation. She was first treated with cytoreductive therapy with hydroxyurea. However, hydroxyurea failed to improve hyperleukocytosis, and on hospital day 25, she presented with subcutaneous bleeding around both eyelids secondary to severe disseminated intravascular coagulation (DIC). On day 26, she developed sudden dyspnea and hypoxia. Chest computed tomography revealed bilateral shadows in the lung, which was highly suggestive of leukostasis of the lungs. She required a respirator. On day 27, chemotherapy comprising idarubicin and cytarabine was started, but on the following day, she developed oliguria, which was suggestive of renal failure due to tumor lysis syndrome (TLS) and sepsis. She required hemodialysis. On day 37, hematological remission was confirmed by bone marrow examination. On day 48 of her hospital stay, she died of multiple organ failure secondary to severe infection and gastrointestinal bleeding.

## 3. Review of Literature and Discussion

The t(7;11)(p15;p15) translocation has been reported to occur not only in AML but also in the acute phase of chronic myelogenous leukemia, chronic myelomonocytic leukemia, and myelodysplastic syndrome [2, 8]. AML-associated translocation is often reported in Asian countries [3–5]. Chou et al. analyzed 493 adult patients with AML in Taiwan and found 11 patients (2.2%) to have the t(7;11)(p15;p15) translocation [3].

As mentioned earlier, the t(7;11)(p15;p15) translocation results in a fusion between NUP98 and HOX [2]. NUP98 is located on chromosome 11p15.5 [9] and belongs to the nucleoporin protein family and forms multisubunit channels in nuclear membranes. These nuclear pore complexes facilitate the transfer of metabolites and molecules between the cytoplasm and the nucleus. NUP98 oncogenes are fused to one of the eight HOX partners, HOXA9, leading to the upregulation of additional HOX genes (HOXA5, HOXA7, HOXA9, and HOXA10) and thereby contributing to leukemogenesis [10, 11]. HOXA9 genes are located on chromosome 7q15 [2]. HOXA9 is a homeodomain-containing transcription factor that plays an important role in hematopoietic stem cell expansion. HOXA9 directly regulates critical downstream genes such as B-cell lymphoma-2 and Ink4a/ARF/Ink4b, which are linked to poor outcomes [10]. Moreover, patients with AML harbor the NUP98-HOXA9 fusion that has been shown to be strongly associated with KRAS and WT1 mutations [3].

The clinical characteristics of patients with AML with the t(7;11)(p15;p15) translocation are reported [3, 4]. In total, 17 patients with the t(7;11)(p15;p15) translocation have been reported, with most patients being relatively young (median age 34 years, range 18–67) and the majority being women (13 of 17 patients). Moreover, most patients presented with the M2-subtype, according to the FAB classification (M2, nine patients; M4, four patients; M5, three patients; M6, one patient). Flow cytometry revealed CD33 (*n* = 17 patients), CD13 and CD117 (*n* = 15 patients), and HLR-DR and CD34 (*n* = 12 patients) antigens [4]. All patients were treated with conventional chemotherapeutic drugs, including anthracycline, cytarabine, and homoharringtonine. The rate of complete remission (CR) was 88.2% (15/17 patients). Two patients underwent allogeneic hematopoietic stem cell transplantation (HSCT) (1 patient was after first remission and the other after relapse) [4]. AML patients with the translocation had poorer OS (median: 13.5 vs. 20 months) and DFS (median: 6 vs. 12 months) than those without the translocation [3].

Harada et al. analyzed 91 AML patients with the t(7;11)(p15;p15) translocation from 1986 to 2014. The 3-year OS and DFS rates after allogeneic HSCT were 40.1% and 37.8%, respectively. Patients who underwent allogeneic HSCT after the second CR were more likely to relapse than those who underwent allogeneic HSCT in the first CR. The 2-year OS and DFS rates of AML patients with the translocation who underwent allogeneic HSCT after the first CR were 60.6% and 56.1%, respectively. These rates were higher than those observed in patients who underwent allogeneic HSCT after the second CR (OS, 37.3%; DFS, 25.0%) [12].

Simple variants of the t(7;11)(p15;p15) translocation have been reported [6, 7]. A 52-year-old man with AML (FAB classification M2) had the t(1;11)(q23;p15), add (7)(q22) karyotype [6]. This case involved chromosome 11p15, indicating its association with NUP98. A 76-year-old woman was diagnosed with AML (FAB classification M2) whose karyotype was a complex t(7;11)-variant, i.e., t(7;11;13;17)(p15;p15;p?;p1?2); this case involved NUP98 [7].

The patient in the present report was a 66-year-old woman with AML; the blast morphology was unclassifiable according to the FAB classification. G-banding of blasts revealed the t(7;7)(p15;p22) translocation, whereas that of T cells revealed a normal karyotype. These findings indicated translocation, which involved chromosome 7p15, indicating its association with HOX. The white blood cell count at the presentation of the present case was high at 97,800 cells/*µ*L. Previous studies have reported that hyperleukocytosis leads to leukostasis, DIC, and TLS, which are associated with early death after the diagnosis of leukemia [13–15]. It is speculated that in the present case, the translocation caused rapid leukemic cell expansion, resulting in hyperleukocytosis, which led to severe DIC, leukostasis of the lungs, and renal failure due to TLS. These complications affected the patient's survival, which was very short: 48 days after admission. Based on this history, the t(7;7)(p15;p22) translocation may cause a rapid expansion of leukemic cells, leading to leukocytosis. This translocation may also be associated with poorer OS as well as t(7;11)(p15;p15) translocation.

## 4. Conclusion

To the best of our knowledge, this is the first report of a patient with the AML with t(7;7)(p15;p22) translocation, a simple novel variant of the t(7;11)(p15;p15) translocation.

## Data Availability

No data were used to support this study.
